# Harnessing TRAIL-induced cell death for cancer therapy: a long walk with thrilling discoveries

**DOI:** 10.1038/s41418-022-01059-z

**Published:** 2022-10-04

**Authors:** Antonella Montinaro, Henning Walczak

**Affiliations:** 1 Centre for Cell Death, Cancer, and Inflammation (CCCI), UCL Cancer Institute, University College London, 72 Huntley Street, London, WC1E 6DD UK; 2 CECAD Cluster of Excellence, University of Cologne, 50931 Cologne, Germany; 3 Center for Biochemistry, Medical Faculty, Joseph-Stelzmann-Str. 52, University of Cologne, 50931 Cologne, Germany

**Keywords:** Cancer, Cell biology

## Abstract

Tumor necrosis factor (TNF)-related apoptosis inducing ligand (TRAIL) can induce apoptosis in a wide variety of cancer cells, both in vitro and in vivo, importantly without killing any essential normal cells. These findings formed the basis for the development of TRAIL-receptor agonists (TRAs) for cancer therapy. However, clinical trials conducted with different types of TRAs have, thus far, afforded only limited therapeutic benefit, as either the respectively chosen agonist showed insufficient anticancer activity or signs of toxicity, or the right TRAIL-comprising combination therapy was not employed. Therefore, in this review we will discuss molecular determinants of TRAIL resistance, the most promising TRAIL-sensitizing agents discovered to date and, importantly, whether any of these could also prove therapeutically efficacious upon cancer relapse following conventional first-line therapies. We will also discuss the more recent progress made with regards to the clinical development of highly active non-immunogenic next generation TRAs. Based thereupon, we next propose how TRAIL resistance might be successfully overcome, leading to the possible future development of highly potent, cancer-selective combination therapies that are based on our current understanding of biology TRAIL-induced cell death. It is possible that such therapies may offer the opportunity to tackle one of the major current obstacles to effective cancer therapy, namely overcoming chemo- and/or targeted-therapy resistance. Even if this were achievable only for certain types of therapy resistance and only for particular types of cancer, this would be a significant and meaningful achievement.

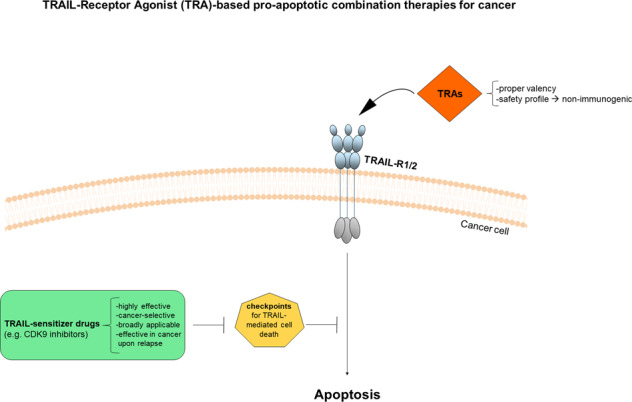

## Facts

- TRAIL kills many cancer cells, but does not induce apoptosis in most normal cell types, a discovery that led to the clinical development of TRAIL-receptors agonists.
- Clinical success of novel TRAIL-R agonists depends not only on increased agonistic activity, but also on their safety profile.
- Combination of highly active and safe TRAs with CDK9 inhibitors is highly effective in many types of cancer and, importantly, also in cancers that exhibit either intrinsic or acquired resistance to current standard-of-care therapies.

## Open Questions

- Can we harness death receptor-induced cell death for effective cancer therapies?
- Which of the second-generation TRAs show a proper valency, a safety profile, and are likely to progress forward in the clinic?
- Will the combination of optimized TRAs with CDK9 inhibitory drugs allow us to introduce in the clinic highly active and safe TRAIL-based pro-apoptotic therapies?

## Introduction

The concept of actively inducing necrosis in tumors was discovered and first applied clinically by William Coley in the late 19th century [1, 2]. However, the advent of chemotherapy at the beginning of the 20th century which, given its chemical nature, could be more reproducibly provided, marginalized this more biologically-based approach which proved more difficult to reproduce. Yet, with the identification of tumor necrosis factor (TNF) – and the coining of its name – as the factor responsible for exerting the anti-tumor activity of Coley’s toxins [3] and the subsequent cloning of TNF, this concept was reinvigorated. However, the purified activity referred to as TNF was only capable of inducing necrosis in select cancers, especially in sarcomas [2, 3], and this activity remained the exception rather than rule. Even more damaging to the concept of using recombinant TNF as a systemic cancer drug was the fact that its systemic application induced lethal inflammation [4]. Consequently, employing TNF as a cancer drug was largely abandoned. Nevertheless, TNF paved the path for the discovery of TNF-like factors for which it was hoped they would fulfill TNF’s promise. The first such factor to be discovered was FasL (CD95L; also known as APO-1L). However, soon after its discovery FasL proved to exert high systemic toxicity and was therefore also abandoned [5, 6]. In search for a weapon to specifically kill cancer cells, another TNF superfamily (TNFSF) member, named TNF-related apoptosis-inducing ligand (TRAIL; also known as Apo2L) was discovered [7, 8]. TRAIL was capable of inducing apoptosis of cancer cells in vitro and in vivo without exerting toxicity [9, 10]. These findings sparked a lot of interest to develop cancer therapeutics within the TNFSF. Thus, apart from a recombinant form of TRAIL itself, various other TRAIL-R agonists (TRAs), have been developed for clinical application. Yet, clinical testing of these first-generation TRAs did not show anticancer activity in patients. Apart from the low agonistic activity of these first-generation TRAs as compared to high-activity forms of TRAIL, some of which had been known since the initial in-vivo studies with this cytokine [9, 11], the other main reason for this failure is likely the resistance of most primary cancer cells to TRAIL-induced apoptosis [11–13]. Consequently, vast efforts have been undertaken to identify, on the one hand, novel, highly active TRAs and, on the other, sensitizing drugs that can break the intrinsic TRAIL resistance of many cancer cells. This review explores the most recent findings on these topics and discusses the most recent advances in how TRAIL resistance might be overcome, hopefully leading to the future development of highly active TRAIL-R agonist-comprising combination therapies within a therapeutic window.

## TRAIL and its receptors

TRAIL is a ~35 kDa protein that shares 65% identity between humans and mice [7], it is expressed in various tissues including spleen, thymus, prostate and lung, and particularly also by cells of the immune system [7]. Structurally, TRAIL is composed of a C-terminus extracellular region comprising the *TNF homology domain* (THD) and an extracellular stalk, which is the part of the protein being cleaved to release the soluble form; a transmembrane helix a short intracellular N-terminus domain [14–16]. The C-terminus domain contains the receptor binding region [15, 17]. Like other TNFSF members, TRAIL forms a homotrimer, however, it is the only protein of the TNF-SF that contains one cysteine residue, Cys230, which allows the interaction and stabilization of the three molecules of TRAIL through the contact of the three cysteines with the zinc atom [18, 19]. Importantly, only the trimeric form of TRAIL is active in killing as mutation of Cys230 was shown to diminish trimerization, rendering the resulting monomeric forms of TRAIL inactive [19]. TRAIL is encoded as a type II transmembrane protein which, similarly to other members of the TNFSF, apart from being present in its membrane-bound version, can also be cleaved from the cell surface through the action of a yet unknown cysteine protease, resulting in the generation of a soluble fragment of ~24 kDa [20]. Interestingly, whilst for CD95L it has been described that only membrane-bound ligand is essential for cytotoxic activity and the soluble form promotes tumorigenesis through non-apoptotic activities [21], the exact role of TRAIL as a soluble form or as a membrane-bound version is currently less clear (Box 1).

In humans TRAIL binds four different membrane-bound TRAIL receptors (TRAIL-Rs) and one soluble receptor. Moreover, TRAIL-Rs can be subdivided in two classes: the ones that contain a full-length intracellular death domain (DD), which is essential for cell death induction, TRAIL-R1 (also known as DR4) [22] and TRAIL-R2 (also known as DR5) [23] [24–29] and the alternative receptors, that are incapable of transmitting an apoptotic signal since they either lack a DD or contain a truncated one, TRAIL-R3 (also known as DcR1) [28–31], TRAIL-R4 (also known as DcR2) [32, 33], respectively and the soluble receptor osteoprotegerin (OPG) [34]. OPG is a conventional soluble receptor for receptor activator of nuclear factor‑κB (NF‑κB) ligand (RANKL) [35], another member of the TNFSF, but has been shown to also bind TRAIL [34].

Following TRAIL binding, TRAIL-R1 and TRAIL-R2 can form both homo- as well as heterotrimeric complexes capable of inducing apoptosis via the same pathway components. These higher-order ligand-receptor complexes can include either trimerization between trimers or contain trimers which are crosslinked to neighboring trimers via dimerization between ligand-opposing receptor interfaces, resulting in a hexameric honeycomb-like structure [11, 36, 37]. TRAIL-R1 and TRAIL-R2, share 58% sequence homology similarity, but the main molecular difference between these two pro-apoptotic receptors is that there is only one splice variant for TRAIL-R1, whereas TRAIL-R2 exists in two isoforms [38]. The long isoform contains 29 additional extracellular amino acids that are rich in threonine, alanine, proline and glutamine (TAPE), and are therefore referred to as the TAPE domain [29]. Importantly, these two receptors differ in their membrane proximal domain (MPD), a short, ten amino-acid-long stretch juxtaposed to the plasma membrane. We recently previously discovered that TRAIL-R2, but not TRAIL-R1, is capable of mediating cancer progression, invasion, and metastasis by cancer cell-autonomous activation of MPD- Ras-related C3 botulinum toxin substrate 1 (Rac1) axis [39]. It is therefore likely that the differences in the MPD enable TRAIL-R2, but not TRAIL-R1 to favor cancer progression. The relative contribution of these two receptors to TRAIL-induced apoptosis in cancer cells is still not fully characterized and appears to be cell type-dependent. In primary lymphoid malignancies, chronic lymphocytic leukemia and some cell lines derived from pancreatic cancer TRAIL-induced killing of cancer cells is mediated via TRAIL-R1 [40–43]. Nevertheless, in some glioma, leukemia, lymphoma and liver cancer cell lines, antibody-mediated TRAIL-R2 activation seems to be sufficient to induce apoptosis [44]. Thus, TRAIL-R1 and TRAIL-R2 exert some unique, but also overlapping functions and further investigations will be required to clearly dissect their respective functions. One possible way to address this question in vivo could be to generate mice expressing these two different human TRAIL-Rs.

TRAIL-R3 and TRAIL-R4 share high identity with the extracellular domains of the death receptors. Whilst TRAIL-R3 is glycosylphosphatidylinositol (GPI)-anchored to the plasma membrane and hence lacks an intracellular domain, TRAIL-R4 only comprises a truncated DD and induces activation of NF-κB upon overexpression [33]. The functions of TRAIL-R3 and -R4 have not been clearly defined, although both receptors have been suggested to act as “decoy” receptors (DcR1 and DcR2) by catching TRAIL molecules and thereby decreasing the probability of TRAIL binding to the apoptosis-inducing receptors [45], but also by negatively affecting TRAIL-death-inducing signaling complex (DISC) formation as TRAIL-R4 has further been proposed to form heterotrimeric complexes with TRAIL-R2 [46]. However, whilst most of these observations were only made in overexpression systems, a recent study supported this notion by showing that TRAIL-R3 and -R4 protected human hepatic stellate cells (hHSC) from TRAIL-induced apoptosis [47]. Importantly, it was shown that especially TRAIL-R4 is the critical physiological regulator of hHSC apoptosis [47].

Regarding their expression, the most widely expressed TRAIL-Rs are TRAIL-R1 and TRAIL-R2 with TRAIL-R3 and TRAIL-R4 being less frequently expressed but quite prominent in immune cells [48]. Importantly, TRAIL was shown to bind TRAIL-R2 with the highest affinity at 37 °C compared to other membrane-bound TRAIL-Rs, indicating that, under physiological conditions, the interaction of TRAIL with TRAIL-R2 might be favored over interaction with any other TRAIL-Rs [49].

Unlike the human TRAIL-R system, mice only express a single DD-containing TRAIL-R (mTRAIL-R, also known as MK) which is highly homologous to human TRAIL-R1 and –R2 and is thus capable of inducing apoptosis [50]. Additionally, two mouse decoy receptors have been described that lack an intracellular DD, namely mDcTRAIL-R1 and mDcTRAIL-R2 [51] as well as the soluble mOPG have been identified in mice [35]. mDcTRAIL-R1 and mDcTRAIL-R2 differ substantially in their amino acid sequence from human TRAIL-R3 and TRAIL-R4 and are incapable of inducing cell death or NF-κB activation upon overexpression and their functions are still largely unknown [51].

Box 1 Forms of TRAIL and their known functions Although the different functions exerted by the soluble form and membrane-bound version of TRAIL in different contexts require further investigation, it has, however, been shown that soluble TRAIL can exert angiogenic activity in soft tissue sarcomas [151]. Recently, a membrane-bound short form of TRAIL, which lacks the THD, has been described to counteract cell death induced by full-length TRAIL in human cancers (reviewed in de Miguel and Pardo [152]). Interestingly, expression of membrane-bound TRAIL on the surface of natural killer (NK) cells can promote killing of cancer cells and contribute to cancer immune-surveillance [153, 154]. Moreover, liposome-bound TRAIL, which mimics native cell-surface expression, shows greater tumor apoptosis-inducing activity than its soluble counterpart [155, 156]. Under physiological condition soluble TRAIL exists in the blood plasma at around 100 pg/ml, a concentration that does not lead to apoptosis in cell lines in vitro [157]. Pro-inflammatory cytokines and lipopolysaccharide (LPS) can induce TRAIL upregulation on the surface of monocytes, macrophages, dendritic cells (DCs) and NK cells [158, 159]. Intracellular and cell surface expression of TRAIL has been found in LPS-activated human monocytes and macrophages and both membrane-bound and soluble TRAIL are involved in the effector mechanisms of activated innate and adaptive immune cells [160]. Interestingly, TRAIL has also been shown to be released associated with exosomes by different immune cells such as DCs, NK and T cells, and to exert not only immunoregulatory functions but also to trigger apoptosis in cancer cells [161] [162, 163]. Additionally, type I IFNs have been shown to boost TRAIL expression on T cells following viral infection, thereby increasing their cytotoxic activity [164]. Intriguingly, soluble TRAIL has been found in the bronchoalveolar lavage from patients with acute respiratory distress syndrome following viral infections [165, 166] and activation of the TRAIL/TRAIL-R pathway has been linked with alveolar epithelial cell death and consequent lung injury [166–168], suggesting that the immune response to viral infections involves the activation of the TRAIL/TRAIL-R system and that this needs to be tightly controlled to prevent damage.

## Signal transduction by TRAIL receptors

The TRAIL/TRAILR system has an important role in the regulation of a variety of biological responses in cancer and normal cells including induction of cell death by apoptosis and necroptosis, as well as initiation of TRAIL-mediated non-cell death-inducing signaling pathways (Box 2).

Box 2 TRAIL-induced non-canonical signaling pathways It has long been puzzling scientists in the field of cell death that cancer cells constitutively expressed TRAIL-R1 and TRAIL-R2 and could therefore be targeted by TRAIL-based apoptotic therapy. Interestingly, data from stage III unresectable non-small cell lung cancer (NSCLC) patients indicated that high TRAIL-Rs expression, specifically TRAIL-R2, positively correlated with increased risk of death [169]. Additionally, high TRAIL-R2 expression positively correlated with markers of malignancy in patients with Kirsten rat sarcoma (KRAS)-mutated cancers [39]. Hence, these observations highlight relevance for a pro-tumorigenic function of the TRAIL-TRAIL-R system. Indeed, binding of TRAIL to TRAIL-R1, -R2 and -R4 has been shown to trigger activation NF-κB [24, 33, 170] a crucial transcription factor involved in several inflammatory and pro-survival pathways. By employing RIPK1- or IkappaB kinase (IKK)γ-deficient cells, TRAIL has been reported to mediate NF-κB activation and thereby promote survival and proliferation of TRAIL-resistant Jurkat cells [171]. Although the initiation of gene-activatory signaling is thought to emanate from complex II, a complex that apart from retaining FADD, caspase-8 and RIPK1 can recruit TRAF2 and IKKγ [149], it has recently been shown that complex I of TRAIL signaling is also capable of initiating gene activation [78] (Fig. 2). Furthermore, LUBAC has been identified as a component of both complex I and complex II and to facilitate the recruitment of IKK complex to both complexes and to limit activation of caspase-8, thereby promoting gene activation and restricting cell death [78] (Fig. 2). Additionally, the TRAIL–TRAIL-R signaling in cancer cells has also been shown to facilitate the accumulation of a tumor-supportive immune environment by triggering an NF-κB-dependent gene activation, which elicited the production of cytokines, most importantly C‑C motif chemokine ligand 2 (CCL2), consequently promoting tumor growth via a CCL2/(C‑C motif) receptor 2 (CCR2) axis [147]. Intriguingly, the non-canonical TRAIL-mediated signaling arm can also be triggered by the MPD of TRAIL-R2, independently of its DD and FADD [39] (Fig. 2). Hence, it would be interesting to study whether blocking the TRAIL–TRAIL-R system could be of therapeutic benefit in cancers that hijack this system to their advantage (reviewed in von Karstedt and Walczak [13]).

### TRAIL signaling towards apoptosis

**Fig. 1:**
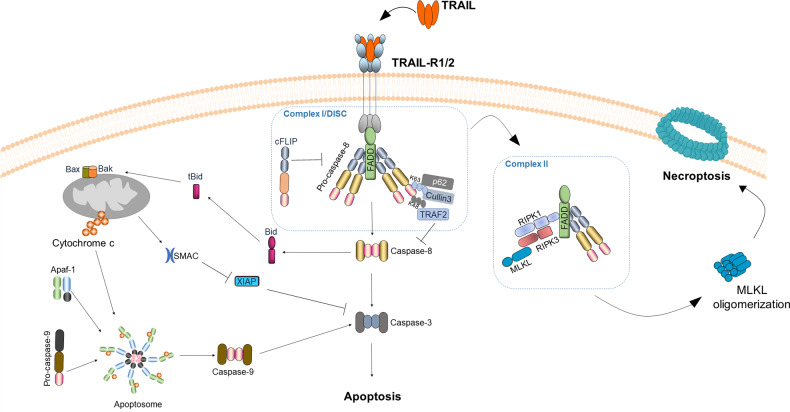
TRAIL-induced cell death signaling pathways. TRAIL-mediated death receptor trimerization enables the recruitment of FADD, which in turn binds to procaspase-8 to form the DISC. TRAIL-Rs, FADD and caspase-8 appear to exist in a 3:1:9 (Receptor:FADD:Caspase) stoichiometry [143]. cFLIP isoforms differentially control TRAIL signaling. Caspase-8 oligomerization and activation can be facilitated by the E3-ligases Cullin-3 which mediates K48/K63 ubiquitination of caspase-8 and subsequent recruitment of the Ub-binding protein p62 to the DISC, allowing p62-mediated aggregation and full activation of the caspase-8caspase-8 [144]. Following ubiquitination of caspase-8, TNFR-associated factor 2 (TRAF2) interacts with caspase-8 at the DISC, down-stream of Cullin3 and it is required for K48-linked polyubiquitination on the large catalytic domain of caspase-8, hence, triggering the proteasomal degradation of this protease and serving as a shut-off timer for the death ligand-mediated apoptosis [145]. In type I cells, DISC-activated caspase-8 is sufficient to activate caspease-3 and trigger apoptosis by the extrinsic pathway. In type II cells full activation of caspase-3 is inhibited by XIAP and therefore activation of the intrinsic apoptosis pathway is essential for full activation of caspease-3. Caspase-8 mediated activation of tBid triggers Bax and Bak to execute mitochondrial outer membrane permeabilization (MOMP). MOMP results in the release of second mitochondria-derived activator of caspase (SMAC), thereby enabling the full activation of caspase-3. Additionally, cytochrome c is also released from the mitochondria and along with adaptor protein apoptosis protease-activating factor-1 (Apaf-1) forms the apoptosome which activates caspase-9, to enable amplification of caspase-3 activation and apoptosis. Upon TRAIL stimulation, following DISC assembly, a secondary cytoplasmatic complex can be formed, known as complex II, which retains the DISC components FADD, caspase-8 and RIPK1. In absence of caspase-8 or when its activity is blocked, RIPK1 recruits RIPK3, which in turn phosphorylates MLKL. Phosphorylated MLKL then oligomerises, which results in the execution of necroptosis.

Binding of TRAIL to its death receptors initiates receptor trimerization leading to the formation of higher-order complexes via the induction of homotrimeric and possibly also heterotrimeric receptor-ligand complexes [11, 36, 37]. The crosslinked receptor trimer initially recruits the intracellular adapter molecule Fas-associated death domain (FADD) via its death domain (DD) to the DD of TRAIL-R1 and/or TRAIL-R2. Subsequently, FADD enables the recruitment of the protease caspase-8 and 10 to its death effector domain (DED) via their respective DEDs resulting in the formation of the DISC, also referred as TRAIL-R-associated complex I (Fig. 1) [52–54]. TRAIL DISC formation enables activation of both caspase-8 and caspase-10 via proximity-induced dimerization and proteolytic cleavage [55, 56]. A structural analysis via electron microscopy revealed how the formation of FADD-nucleated tandem DED (tDED) helical filaments is essential to orientate the procaspase-8 catalytic domains, allowing their activation via anti-parallel dimerization [57]. Once activated, caspases 8 and 10 are released into the cytosol where they cleave and activate downstream substrates such as caspase-3. Although caspase-10 undergoes similar cleavage kinetics as caspase-8, the ability of caspase-10 to compensate for the loss of caspase-8 remains controversial. Intriguingly, it has recently been shown that caspase-8 is required upstream of caspase-10 and that caspase-10 acts as a negative regulator of CD95L-induced apoptosis [58]. Thus, caspase-8 is critical for DISC formation and death-ligand-induced apoptosis. In the so-called type I cells the activation of caspase-8 at the DISC is sufficient to induce a robust activation of caspase-3 and consequently of apoptosis through the extrinsic pathway [59, 60]. Alternatively, in so-called type II cells, caspase-8 activation is often insufficient and requires further cell-intrinsic amplification via cross-signaling to the mitochondria [61]. This is achieved via caspase-8-mediated cleavage of BH3-interacting domain death agonist (Bid) to generate truncated Bid (tBid) [61] which interacts with B-cell lymphoma 2 (Bcl-2)-associated X (Bax) and Bcl-2 homologous antagonist killer (Bak) to execute mitochondrial outer membrane permeabilization (MOMP) [62]. This results in release of several pro-apoptotic factors such as cytochrome C and the second mitochondria-derived activator of caspases (SMAC), a natural antagonist for X-linked inhibitor of apoptosis (XIAP), which is the key discriminator between type I and type II cells [63]. SMAC then counteracts XIAP, while cytochrome associates with the adaptor molecule apoptotic protease activating factor 1 (Apaf1) to assemble a platform for the intracellular initiator caspase-9 to form a caspase-activating multi-protein complex referred to as the apoptosome (Fig. 1), thus allowing full cleavage and activation of effector caspases including caspase-3 [64]. These activated effector caspases are the driving force behind cleavage of a variety of cellular protein substrates which ultimately results in cleavage of the inhibitor of caspase-activated DNAse (iCAD) and, consequently, activation of caspase-activated DNAse (CAD) finally leading to deoxyribonucleic acid (DNA) fragmentation and membrane blebbing the typical hallmarks of apoptosis [65, 66].

### Caspase-independent cell death induced by TRAIL

**Fig. 2:**
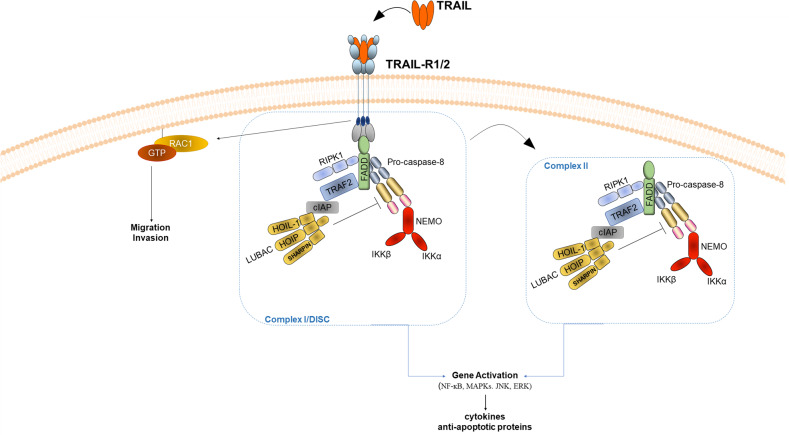
Non-canonical TRAIL signaling. In complex I and complex II downstream of FADD, caspase-8, TRAF2 and cIAP1/2, the linear ubiquitin chain-assembly complex (LUBAC) limits caspase-8 activation and enables the recruitment of the IKK complex to both complexes promoting gene activation and cytokine production. When caspases are inhibited RIPK1 can induce NF-κB [146]. FADD is essential for the formation of both complex I and complex II it is therefore also required for TRAIL-induced gene-activatory signaling [78, 147]. Caspase-8 presence, but not its activity is crucial for TRAIL-mediated gene activation [147, 148]. Besides NF-ĸB, TRAIL is also implicated in the activation of mitogen activated protein kinases (MAPKs), including c-Jun N-terminal kinase (JNK), p38 and extracellular regulated kinase (ERK)1/2, which control central physiological processes such as gene expression, cell proliferation and inflammation [149, 150]. Additionally, independently of FADD the membrane-proximal domain (MPD) of TRAIL-R2 can activate Rac1 and promote progression, invasion and metastasis [39].

Apart from apoptotic cell death, TRAIL can mediate a programmed form of caspase-independent cell death known as necroptosis. Necroptosis is a tightly regulated process mediated by the action of receptor-interacting protein kinase (RIPK)1, RIPK3 [67–69], which form the core of the necrosome complex, as well as the executer mixed linkage kinase domain like pseudokinase (MLKL) [70–72], resulting in membrane bursting, cellular leakage [73]. Even though necroptosis was originally described to be induced by TNF, soon thereafter it was found that cell death triggered by CD95L and TRAIL can also be necroptotic [74, 75] (reviewed in Vandenabeele et al. [76]). It was further shown that mainly the kinase activity of RIPK1 was required for mediating necroptosis via identification of the specific RIPK1 inhibitor necrostatin, capable of blocking this kind of cell death [77]. TRAIL-induced necroptosis is thought to originate from complex II, a TRAIL-R-devoid cytosolic complex which acts as a DISC which retains FADD, caspase-8 and also contains RIPK1 [78] (Fig. 1). When caspase-8 is absent or its activity is hampered, necroptosis is unleashed [79–81]. Since caspase-8 has been identified to inhibit necroptosis by cleavage of RIPK1 and RIPK3, and since FADD is required for caspase-8 activation, caspase-8 and/or FADD deficiency facilitate necroptotic cell death. Accordingly, embryonic lethality of caspase-8 and FADD knockout mice, which succumb to enhanced necroptosis, could be reversed by co-ablation of RIPK3 or RIPK1 [81–85]. To add another level of complexity, TRAIL–TRAIL-R signaling is also tightly regulated by various types of ubiquitination events (reviewed in Lafont et al. [86]). The E3 known as “linear ubiquitin chain assembly complex” (LUBAC) is composed of SHARPIN, HOIL-1 and the catalytic component HOIP and restricts TRAIL-induced necroptosis by limiting the formation of the necroptosis-mediating complex [78] (Fig. 2). Nevertheless, how exactly the different ubiquitination events regulate the TRAIL-induced necroptosis-mediating complex formation and function requires further investigation. Since necroptotic cell death promotes inflammation and immune activation it would be appealing to further test how TRAIL-induced necroptosis will affect tumor immunogenicity and treatment outcome especially in the context of cotreatment with immune checkpoint inhibitors.

## Mechanisms of TRAIL resistance in cancer cells and possible therapeutic interventions

Cancer cells employ various mechanisms to escape apoptosis (Box 3). Several checkpoints for TRAIL-mediated cell death have been identified to date and are in place at different levels along the extrinsic apoptosis pathway including upregulation of the cellular FLICE-like inhibitory protein (cFLIP) to block apoptosis at the DISC, upregulation of Bcl-2 family members as well as inhibitor of apoptosis proteins (IAPs) to block apoptosis at the mitochondrial level. Although a comprehensive elucidation of all the mechanisms driving TRAIL resistance in cancer cells has yet to be fully characterized, most of the biological mechanisms and steps responsible for TRAIL resistance in cancer cells have been identified and have led to the development of several TRAIL-sensitizing strategies to overcome this resistance.

Box 3 Additional mechanisms responsible for resistance to TRAIL-induced apoptosis Mutations in the death receptors have also been proposed to be at least partially responsible for resistance to TRAIL-induced apoptosis in non-Hodgkin’s lymphoma [172], metastatic breast [173], lung as well as head and neck cancers [174]. Post-translational modifications, in particular glycosylation, of TRAIL-R1/TRAIL-R2 have also been found to regulate TRAIL’s ability to trigger apoptosis (reviewed in Micheau [175]). Furthermore, intracellular and nuclear localization of TRAIL-Rs has also been suggested to mediate resistance to TRAIL-induced apoptosis and to support tumor progression (reviewed in Bertsch et al. [176]). Interestingly, endogenous TRAIL-R2 has recently been shown to promote proliferation of pancreatic cancer cells, in a ligand-independent manner, via inhibiting the processing of let-7 miRNA [177]. Additionally, alternative resistance mechanisms to TRAIL-induced apoptosis have also been shown, such as cell-to-cell variability in initial TRAIL sensitivity within clonal cell populations [178]. According to this model, constant TRAIL stimulation can lead to selection of pre-existing high Bcl-2-expressing cells instead of upregulation of these proteins upon persistent TRAIL exposure [178]. Furthermore, extrinsic apoptosis resistance including TRAIL resistance can be acquired through persistent immune-mediated selective pressure in human tumors as it has recently been shown that inactivating caspase-8 mutations are frequently found in solid tumor biopsies and positively correlated with immune cytolytic activity [179]. Thus, loss of the extrinsic apoptosis pathway could be a general mechanism employed by tumors to escape cytolytic immune cells [179].

### Checkpoints at the DISC

cFLIP, which shares high sequence homology with the initiator caspases 8 and-10 and contain a FADD-binding DED [87, 88], controls DISC assembly and activation. It exists in three main splice variants a long isoform cFLIP_L_, which strongly resembles full-length procaspase-8, but it is catalytically inactive, and two short isoforms cFLIP_S_ and cFLIP-Raji (cFLIP_R_), which are a truncated version of procaspase-8 [88, 89]. Whilst the two short isoforms function as inhibitors of procaspase-8 activation, cFLIP_L_ can function as pro-apoptotic as well as anti-apoptotic regulator of procaspase-8 activation depending on the extent of death receptor stimulation and its expression level [87, 90]. Although it was long thought that cFLIP inhibits caspase-8 activation in a dominant-negative manner by competing with it for binding to FADD through the formation of heterodimers with procaspase-8 [87, 91, 92], it has recently been shown that the binding of cFLIP_L/S_ to FADD is a co-operative and hierarchical procaspase-8-dependent process [93]. Importantly, the procaspase-8:cFLIP co-operative binding model explained how the balance of c-FLIP_L_/_S_ to procaspase-8 is vital in determining signaling for death or survival [93]. According to this model, whilst only a limited amount cFLIP_S_ is required to block both DISC-mediated oligomerization and activation of procaspase-8, very high levels of c-FLIP_L_ are needed to inhibit DED-mediated procaspase-8 oligomer assembly [93]. Moreover, mechanistically cFLIP_S_ disrupts caspase-8 activation by preventing caspase-8 filament elongation via steric hindrance of the canonical tandem DED binding site, consequently altering the architecture of the FADD:Caspase-8 complex [57]. Interestingly, cFLIP has been shown to act as a negative regulator of RIPK1, caspase-8 and HOIP recruitment, thereby restricted accumulation of linear ubiquitination and IKK activation in complex I of TRAIL signaling [78]. Apart for regulating apoptosis cFLIP also plays a crucial role in the regulation of necroptosis [94]. By cleaving RIPK1 and RIPK3, procaspase-8:cFLIP_L_ heterodimer can prevent necroptosis, whereas by completely preventing caspase-8 activation cFLIP_S_ restricts apoptosis but promotes necroptosis [79–81]. Thus, since all cFLIP isoforms are major regulators of the different TRAIL-induced signaling outputs, they may represent a promising target for TRAIL-based pro-apoptotic therapies.

### Bcl-2 and IAP family

In cells that require a signal-amplifying loop via involvement of the mitochondria to trigger apoptosis, the balance between pro-apoptotic effectors (Bax and Bak), pro-apoptotic initiators (Bim, PUMA, Bid, NOXA, Bmf, Bik, and HRK) and pro-survival (Bcl-2, Bcl- xl, Mcl-1, A1 and Bcl- w) Bcl-2 family members is crucial to dictate cell fate [95]. The pro-apoptotic initiator proteins, which are also called “BH3-only” proteins, can be upregulated transcriptionally or post-transcriptionally in response to cellular stress or oncogenic activation and trigger apoptosis by either binding the pro-survival Bcl-2 members or by directly activating Bax and Bak, which are kept in check by pro-survival Bcl-2 proteins [96]. Consequently, cancer cells can acquire resistance to TRAIL-induced apoptosis by upregulation of these pro-survival proteins as well as loss of function of pro-apoptotic proteins [97–101]. Decrease in the pro-apoptotic/pro-survival Bcl2 ratio, mainly due to upregulation of pro-survival Bcl-2 family members, is observed in several human cancers [95, 96]. Therefore, cancer cells that are highly dependent on pro-survival proteins such as Bcl-2 and/or Mcl-1 are less likely to respond to TRAIL-induced apoptosis. Several small molecules have been developed to specifically inhibit pro-survival Bcl-2 family proteins by mimicking the function of the BH3-only proteins and which are therefore termed BH3 mimetics [102]. A number of clinical trials are currently ongoing to evaluate BH3 mimetics as monotherapy as well as in combination regimens, however, the Bcl-2-selective inhibitor ABT-199 (also known as venetoclax) is the only BH3 mimetic that has been approved by the Food and Drug Administration (FDA) to date for the treatment of hematological malignancies [103]. One of the main limitations of the clinical development of additional BH3 mimetics is the on-target toxicity due to the inhibition of pro-survival proteins crucial for many physiological functions [102], therefore, the identification of a therapeutic window and/or possible combination therapies to reduce toxicity and achieve long and stable response would be essential for the further clinical advance of these compounds [104].

Apart from dysregulation of Bcl-2 family members, TRAIL resistance can also be mediated by overexpression of IAPs. These group of anti-apoptotic proteins are highly upregulated in different cancer types to favor tumorigenesis and treatment resistance [105]. Amongst the IAPs family, XIAP, cIAP1, and cIAP2 act as E3 ubiquitin ligase thanks to their really interesting new gene (RING) domain, which mediates ubiquitination of several cell death modulator and effector proteins, therefore regulates TRAIL-induced cell death or gene activation [106–108]. Whilst cIAP1 and cIAP2 contain a caspase­ recruitment domain, they are unable to directly inhibit caspases; hence their ubiquitin-protein ligase activity is crucial to regulate cell survival and apoptosis resistance [109–111]. Accordingly, cIAP1/2-mediated inactivation of caspases occurs in a Ub-dependent manner by targeting caspases, such as caspase-3 and -7 for proteasomal degradation [112]. XIAP is the only member of the IAP family capable of directly inhibiting caspase-3, caspase-7 and caspase-9 [113] and its expression levels are critical to classifying cells as type I or type II regarding death ligand-mediated apoptosis [63]. Importantly, as mentioned above, since cytosolic SMAC can neutralize XIAP and releases caspases from their XIAP-imposed inhibition, several small-molecule inhibitors that mimic SMAC, and which are therefore referred as SMAC mimetics, have been designed to antagonize IAP proteins and promote cell death [114]. At least eight different SMAC mimetics have been tested in clinical trials so far and/or are currently assessed in phase I/II trials in solid tumors or hematological malignancies. However, all of these compounds, although well tolerated, exhibited limited activity in monotherapy, thus different combination regimens are being pre-clinically and clinically evaluated, importantly including combinations with TRAs [111].

### The use of small molecule inhibitors as TRAIL-sensitizing agents

**Fig. 3:**
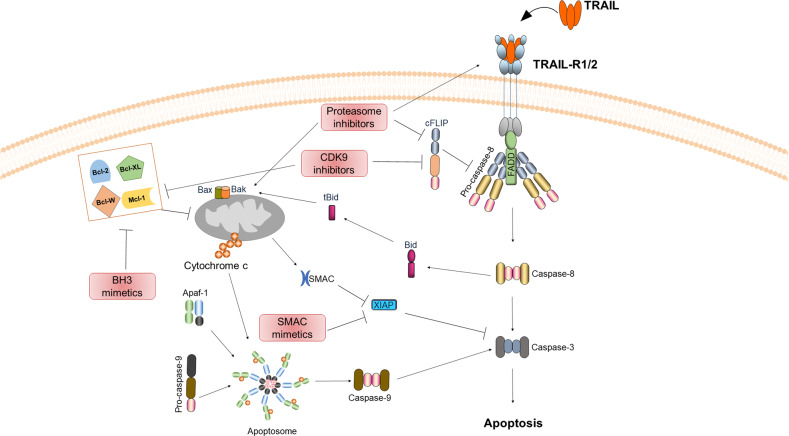
Most promising TRAIL-comprising combination therapies as an effective means of treating cancers. Schematic presentation of the mechanism of action of the combinations of TRAIL with inhibitors of the proteasome, SMAC mimetics, BH3 mimetics or inhibitors of cyclin-dependent kinase 9 (CDK9).

One of the main shortcomings in the clinical development of TRAs has been the intrinsic or acquired resistance of primary cancer cells to TRAIL-induced apoptosis (reviewed in von Karstedt et al. [13]). As described before, resistance can occur through the dysregulation of several modulators of the TRAIL-induced apoptosis pathway such as c-FLIP, XIAP, Bcl-2 and Mcl-1. Hence, efforts have focused on identifying therapeutic strategies which use TRAIL – or a different TRA – as the trigger of extrinsic apoptosis in combination with other therapeutic approaches, mostly small molecule inhibitors that target the above-mentioned checkpoints, thereby aiming to sensitize cancer cells to TRAIL-induced apoptosis in order to obtain a maximum therapeutic window with little to no toxicity. To date, various TRAIL-sensitizing strategies for specific cancer types have been identified (Box 4), however, their safe applicability and/or in vivo efficacy have not been verified. Therefore, in this section we will focus on the most promising, combination treatment strategies identified to date that are aimed at therapeutically overcoming TRAIL resistance. These include combinations of TRAIL with (i) proteasome inhibitors, (ii) BH3 and SMAC mimetics drugs, or (iii) inhibitors of cyclin-dependent kinase 9 (CDK9) (Fig. 3).

The proteasome inhibitor Bortezomib, an FDA-approved drug for the treatment of relapsed and refractory multiple myeloma, has been shown to sensitize different cancer cells to TRAIL-induced apoptosis [115]. The mechanism of action of Bortezomib is complex and has been revealed gradually. In hepatoma, colon carcinoma, pancreatic cancer and primary human esthesioneuroblastoma cells, Bortezomib-mediated sensitization to TRAIL, within the therapeutic window, has been demonstrated to be mediated by enhanced TRAIL DISC formation, heightened caspase-8 presence and cFLIP_L_ downregulation at the DISC and upregulation of TRAIL-R1/TRAIL-R2 [116–118]. In NSCLC bortezomib-induced TRAIL sensitization has been shown to be at least partially depended on NF-κB suppression [119]. At the mitochondrial level, proteasome inhibition can affect the expression of different pro and anti-apoptotic Bcl-2 family proteins and thereby impact TRAIL sensitization [115]. Prolonged proteasome inhibition has been shown to lower the threshold for TRAIL-induced apoptosis via upregulation of different BH3-only proteins [120–122]. Recently, proteasome inhibition has also been proposed to trigger cell death in Bax/Bak-deficient colon cancer cells via the formation of cytosolic TRAIL-R2 containing caspase-8 activation complexes in a death ligand-independent manner [123], however a contribution of endogenously secreted TRAIL cannot be fully excluded. Furthermore, proteasome inhibition can bypass the amplification loop via the mitochondrial apoptotic pathway as Bortezomib-treated Bax/Bak-deficient cancer cells can still be sensitized to TRAIL-induced cell death probably via bortezomib-mediated enhanced DISC formation and stabilization of caspase-8 [116]. All these promising preclinical results and the identification of a therapeutic window to safely combine TRAs with proteasome inhibitors have led to the initiation of two clinical trials (NCT00315757, NCT00791011), yet no additive therapeutic benefits were observed when Bortezomib was combined with first-generation TRAs [124]. The poor agonistic activity of the TRAs employed in these studies might explain the lack of synergy observed in the clinic.

Another promising TRAIL-sensitization strategy comprises the combination of TRAIL with SMAC-mimetics. This combination has been extensively tested both in vitro and in vivo in a variety of cancer entities and exhibited broad preclinical activity (reviewed in Fulda [114]). Thus, a phase 1b clinical trial has been undertaken to clinically determine the maximum tolerated dose and the clinical response of the combination of Birinapant, the clinically most advanced SMAC mimetic, with first-generation TRAs. So far, this combination appears to be well tolerated (NCT01940172) [125]. However, results on potential additive therapeutic benefit are not available yet.

Furthermore, the targeting of the mitochondrial arm of the TRAIL apoptosis pathway by employing BH3 mimetic drugs as a means to overcome TRAIL resistance has also been investigated. BH3 mimetics showed encouraging preclinical activity as TRAIL sensitizers in different cancer types [126–130]. However, the safety of the clinical applicability of this combination has yet to be explored in patients as additional studies are required to establish a therapeutic window for the combinatorial application of BH3 mimetics and TRAs.

Other than combining TRAIL with drugs that directly target crucial roadblocks of the TRAIL-induced apoptosis pathway, TRAIL has also been combined with small-molecule kinase inhibitors as cancer cells are highly dependent on different kinase signaling pathways to favor tumorigenesis and escape cell death [131]. The most potent TRAIL-sensitizing strategy discovered to date consists in the combination of TRAIL with the inhibition of CDK9 [132, 133]. CDK9-inhibitory drugs, which target both CDK9 isoforms [134] (42 kD and 55 kDa), sensitized a panel of TRAIL-resistant NSCLC cell lines, irrespectively of their mutation status, to TRAIL-induced apoptosis by concomitant downregulation of two anti-apoptotic factors, Mcl-1 and cFLIP [132]. Importantly, primary human hepatocytes did not succumb to the same treatment regime, defining a considerable therapeutic window. Yet, it remained to be determined whether the efficacy of this potent cancer-selective combination would be broadly applicable and highly effective across many cancer types, and whether it would also demonstrate therapeutic efficacy in cancer upon relapse. Recently, we embraced the challenge of answering these open questions aiming to identify a treatment regime that could tackle one of the major obstacles to effective cancer therapy, namely, intrinsic and acquired resistance to chemo- and targeted therapy. Intriguingly, the combination of TRAIL with CDK9 inhibition provides for a pro-apoptotic therapy which is not only broadly applicable but also capable of broadly bypassing cancer therapy resistance, importantly independently of whether the resistance is primary or acquired and, when it is acquired, independently of how the resistance was achieved [133]. Interestingly, this combination was highly effective in clinically relevant models that have been shown to predict clinical outcomes in patients, importantly without causing prohibitive or noticeable untoward effects. Mechanistically, this was achieved through an unprecedented increase in mitochondrial apoptotic priming, irrespective of whether the cancer cells were sensitive or resistant to chemo- or targeted therapy. Thus, the combination of optimized TRAs with CDK9 inhibitory drugs, of which many are under clinical evaluation, may bear the potential to bypass therapy resistance in many types of cancer. Additionally, since one of the possible failures of the clinical trials conducted so far with TRAs is the lack of (a) suitable biomarker(s) for patient stratification, identifying patients more – or less – likely to respond to a TRA-comprising therapy, and since CDK9 was found to be highly expressed in cancer tissue compared to the healthy counterpart [133], high CDK9 expression could be considered as an inclusion criterion for therapies that comprise the combination of effective TRAs with drugs capable of inhibiting CDK9. Interestingly, by employing a versatile in-vivo target validation platform, it has recently been shown that indeed CDK9 inhibition is not only a promising target in various cancer types, but that it can also be safely applied within a therapeutic window [135].

Box 4 Targeting pro-survival pathways to sensitize cancer cells to TRAIL-induced apoptosis Recent advances in the molecular characterization of human cancers have identified several recurrent alterations that act as oncogenic drivers. The most commonly activated oncoproteins in human cancers include, amongst others, RAS [180] and RAF [181] proteins, MYC [182] as well as phosphoinositide 3-kinases (PI3Ks) [183]. Oncogenic mutations in these proteins result in constitutive activation of different signaling pathways such as mitogen activated protein kinase (MAPK)- extracellular regulated kinase (ERK), PI3K-AKT-mammalian target of rapamycin (mTOR) and PI3K-AKT-NF-κB pathways, which promote cell survival, proliferation, migration and invasion [184, 185]. Interestingly, resistance to TRAIL-induced apoptosis has also been linked to dysregulation of these pathways, and consequently, inhibition of different kinases such as AKT and PI3K has been shown to induce TRAIL sensitization [186, 187]. In ovarian cancer cells, increased activity of the PI3K-AKT pathway can protect cells from TRAIL-induced apoptosis [188, 189]. Inactivation of the MAPK-ERK pro-survival pathway has been shown to be responsible for detachment-induced sensitization of skin carcinoma cells to TRAIL [190]. Moreover, activating mutations in the PI3KCA gene, which encodes the catalytic p110α subunit of PI3K, have been proposed to render colorectal cancer cells resistant to TRAIL-induced cell death [191]. Different mechanism of actions by which inhibition of these pro-survival pathways promote TRAIL sensitization have been proposed and shown to differ not only according to the cancer cell type studied, but also, more importantly, according to the target specificity of the kinase inhibitors employed. Thus, studies that lack further target validation of the small kinase inhibitors employed need to be considered with caution e.g., the small molecule inhibitor PIK-75, thought to target specifically p110α [192], was later shown to sensitize NSCLC cell lines to TRAIL exclusively via inhibition of CDK9 [132].

## Clinical development of highly potent TRAIL-receptor agonists

The discovery that TRAIL can induce apoptosis selectively in cancer cell without harming normal cells and tissues has led to the clinical development of several TRAs. To date several clinical studies have been undertaken to evaluate TRAs anti-tumoral potential in patients, however, none of these studies reveal therapeutic benefit (reviewed in Lemke et al. [12]). The short half-life, the limited agonistic activity of the molecules chosen for clinical evaluation, the problem of using bivalent antibodies without crosslinking to target a receptor system which requires trimerization are all crucial factors responsible for the clinical failure of first-generation TRAs [12, 13]. Accordingly, recent studies have experimentally and structurally shown that stabilization of higher-order DISCs is required and sufficient to safely sensitize cancer cells to TRAIL-induced apoptosis [11, 37]. Specifically, it was demonstrated the clinically used TRAIL-R2-specific antibody AMG655 and recombinant, non-tagged TRAIL (e.g., Dulanermin), both of which show only little activity in killing cancer cells in monotherapy, when combined they synergized in the killing of cancer cell lines derived from a variety of cancer entities including ovarian and lung cancer [11, 37]. Importantly, together they were approx. as active as recombinant isoleucine zipper-tagged TRAIL (iz-TRAIL) [11, 37]. Moreover, as noted above the lack of patient stratification in respect to possible biomarkers of response, the resistance of many cancers to monotherapy with TRAs as well as the potential immunogenicity of the novel TRAs are additional factors responsible for the failure of clinical trials conducted so far with TRAs (reviewed in von Karstedt et al. [13]). Whilst all the different aspects that contribute to the clinical failure of first-generation TRAs-based approaches have been comprehensively reviewed elsewhere [12, 13, 136], we will now discuss the progress on the development of second-generation TRAs-based treatment approaches and their progresses in the clinic.

### Recombinant forms, cell-based therapies, fusion proteins

#### TRAIL-trimer fusion protein

A fully-human TRAIL-trimer fusion protein, SCB-313, has been developed by in-frame fusion of human C-propeptide of α1collagen (Trimer-Tag) to the C-terminus of human TRAIL resulting in a disulfide bond-linked homotrimer [137]. In preclinical studies TRAIL-trimer fusion proteins have shown increased bioactivity and a safely profile as well as improved pharmacokinetics and enhanced pharmacodynamic activity [137]. So far, there are five phase I clinical trials ongoing with SCB-313 for the treatment of peritoneal malignancies, malignant pleural effusions, malignant ascites and peritoneal carcinomatosis (Table 1). Results of these trials will show whether indeed the promising findings observed in pre-clinical settings hold true in patients.

**Table 1:** Clinical trials with second-generation TRAs.

TRAIL-R agonists	Setting	Cancer	Phase	Clinical trial identifier
SCB-313	Monotherapy	Malignant Pleural Effusion	I	NCT04123886
SCB-313	Monotherapy	Malignant Ascites	I	NCT04051112
SCB-313	Monotherapy	Malignant Pleural Effusions	I	NCT03869697
SCB-313	Monotherapy	Peritoneal Malignancies	I	NCT03443674
SCB-313	Monotherapy	Peritoneal Carcinomatosis	I	NCT04047771
MSCTRAIL	Monotherapy	Adenocarcinoma of Lung	I/II	NCT03298763
ABBV-621 /APG880	Combination therapy (Venetoclax, FOLFIRI, Bevacizumab)	Advanced Solid Tumors Hematologic Malignancies	I	NCT03082209
ABBV-621 /APG881	Combination therapy (Bortezomi, Dexamethasone)	Multiple Myeloma	I	NCT04570631
INBRX-109	Monotherapy	Conventional Chondrosarcoma	II	NCT04950075
INBRX-109	Combination therapy (Carboplatin, Cisplatin, Pemetrexed, 5-fluorouracil, Irinotecan, Temozolomide)	Solid Tumors, Malignant Pleural, Mesothelioma, Gastric Adenocarcinoma, Colorectal Adenocarcinoma, Sarcoma, Pancreatic Adenocarcinoma, Ewing Sarcoma, Chondrosarcoma	I	NCT03715933
GEN1029 (HexaBody®-DR5/DR5)	Monotherapy	Colorectal Cancer, Non-small Cell Lung Cancer, Triple Negative Breas Cancer, Renal Cell Carcinoma, Gastric Cancer, Pancreatic Cancer, Urothelial Cancer	I/II	NCT03576131
IGM-8444	Combination therapy (FOLFIRI, Bevacizumab, Birinapant, Venetoclax)	Solid Tumor, Colorectal Cancer, Gastric Cancer, Non Hodgkin Lymphoma, Non-Small Cell Lung Cancer, Sarcoma, Chondrosarcoma, Small Lymphocytic Lymphoma, Chronic Lymphocytic Leukemia	I	NCT04553692

#### Nanoparticle (NP)-based formulations of TRAIL

Several formulations of TRAIL using NP-based methods, including liposomes, albumin and polymeric NPs have been developed and are currently being established with the aim to increase stability, cancer-specific delivery and/or concomitant delivery of TRAIL with other drugs, such as TRAIL-sensitizer molecules (reviewed in de Miguel et al. [136]). TRAIL can be integrated in NPs either by surface attachment, mimicking physiological membrane-bound protein, or by encapsulating TRAIL inside the NPs to favor its constant and stable release. Although NP-based formulations of TRAIL showed encouraging preclinical results [136] they have not yet undergone clinical testing.

#### MSCTRAIL

Another novel TRAIL delivery approach consists in the development of mesenchymal stromal cells (MSCs) genetically modified to express TRAIL (MSCTRAIL). MSCTRAIL have shown increased half-life, tumor-specific delivery, and anti-cancer activity of TRAIL in preclinical settings [138]. Thus, a phase I/II clinical trial has been lunched and it is currently ongoing to evaluate the safety and anti-tumor activity of MSCTRAIL in addition to chemotherapy in metastatic NSCLC patients (Table 1).

#### Single-chain TRAIL-receptor-binding domain-based agonists

This class of agents has been developed with the aim of maximizing receptor clustering independently of fragment crystallizable (Fc) gamma receptor (FcγR)-mediated cross-linking. The hexavalent agonistic fusion proteins ABBV-621 (also known as APG880), which derives from its prototype APG-350 [139], it is composed of two sets of trimeric native single-chain TRAIL receptor binding domain monomers linked to a human immunoglobulin G1 (IgG1)-Fc mutant domain designed to enable its binding to all FcγRs and the complement component C1q [129]. ABBV-621 has demonstrated promising therapeutic activity in patient-derived xenograft models, both in monotherapy and in combination therapies [129]. A recently completed clinical phase I trial showed the safety, tolerability and potential anti-tumor activity of ABBV-621 in combination with venetoclax in patients with relapsed/refractory hematologic malignancies [140]. Currently, patients with relapsed or refractory multiple myeloma are recruited to evaluate the efficacy and dosage range of ABBV-621 in combination with bortezomib and dexamethasone (Table 1).

### Multivalent-based antibodies

To overcome the limitation of the bivalent nature of the first-generation TRAIL-R-agonistic antibodies novel multivalent TRAIL-Rs antibodies have been developed with the aim of favoring the formation of stable high-order complexes.

#### Tetravalent nanobodies

The tetravalent nanobody TAS266 was the first of this category to enter the clinic. TAS266 is an agonistic multivalent nanobody based on four high affinity single variable domains (VHH) derived from heavy chain antibodies, occurring naturally in camelids, designed to targets TRAIL-R2 [141]. Since each VHH can bind to one TRAIL-R2 molecule, TAS266 can potentially cluster four TRAIL-R2 or bridge two TRAIL-R2 trimers, leading to efficient DISC formation and apoptosis induction as compared to bivalent antibodies. However, the first-in-human study of TAS266 had to be terminated due to acute liver toxicity, possibly due to preexisting antidrug antibodies (ADAs) which could have promoted an increased clustering of the receptors and consequent amplification of the agonistic activity of TAS266 [141]. The results of this clinical trial have clearly highlighted that the potential for immunogenicity must be taken into consideration when designing a biotherapeutic, and that this is particularly true when designing a novel TRAIL-R agonist. They also suggest TRAs need to have a proper valency, possibly best mimicking the one exerted by cell surface-expressed TRAIL. Consequently, a novel tetravalent nanobody, INBRX-109, engineered to eliminate recognition by pre-existing ADAs to lower the potential for hyper-clustering, has been developed and has entered the clinic (https://inhibrx.com/inbrx-109/, Table 1). Phase I and II studies for INBRX-109 in solid cancers are currently enrolling and results of these trials will show the potential stately profile and clinical activity of this compound.

#### Hexabodies

This class of therapeutics have been developed to overcome the full FcγR-mediated antibody crosslinking dependence of conventional TRAIL-R-targeting antibodies by allowing antibody hexamer formation after target binding. The HexaBody-DR5/DR5 (also known as GEN1029) comprises a mixture of two noncompetitive TRAIL-R2-specific IgG1 antibodies, each with a single point mutation in the Fc domain to facilitate intermolecular Fc:Fc interactions, thereby improving hexamer formation upon binding to TRAIL-R2 [142]. First preclinical results obtained with this novel TRA were promising [142], yet a phase I/II clinical trial launched to evaluate the safety of HexaBody-DR5/DR5 in patients with solid tumors has recently been terminated (Table 1). Although the reason for the premature termination of this study has not been revealed yet, the potential toxicity of this hexabody compound cannot be excluded.

#### Pentameric IgM-based TRAs

Since IgM normally exists as a pentamer and since they can effectively bind up to ten repetitive epitopes and low expressing antigens, they provide an intriguing platform to develop novel TRAs able to induce efficient TRAIL-Rs multimerization [142]. IGM-8444 is the first IgM-based TRAs to enter the clinic (Table 1). The preclinical results obtained in solid and hematologic malignancies showed promising anti-tumor activity of IGM-8444 alone or in combination with anticancer agents, however, the potential hepatoxicity was only evaluated in in vitro settings [142]. Thus, the results from this clinical trial will indeed show whether this class of therapeutics can be safetly administered and can provide robust anti-cancer activity.

## Conclusions and perspectives

For the last thirty years the main question within the extrinsic apoptosis field has been how to target the death receptor arm of the apoptosis pathway to introduce in the clinic highly active anti-cancer therapies. When TRAIL was identified and found to be able to induce apoptosis selectively in cancer cells both in vitro and in vivo, in contrast to TNF and FasL, the hope was high. However, when this concept was tested in the clinic it failed. The reasons for this are (*i*) that first-generation TRAs that were tested in the clinic were poor agonists, (*ii*) that certain second-generation TRAs that entered the clinic showed toxicity due to immunogenicity, and (*iii*) that primary cancers are mostly resistant to TRAIL monotherapy, meaning that this resistance had to be overcome to achieve an effective therapy. Whilst lot of efforts are being made to optimize the agonistic activity and safety of novel TRAs, the identification of suitable TRAIL sensitizers could represent the main contributor towards the clinical success of TRAIL-based pro-apoptotic therapies. The recent discovery that combining highly active TRAs with CDK9 inhibitors are broadly applicable and highly effective, importantly in a cancer-selective manner and also in cancers that exhibit either intrinsic or acquired resistance to current standard-of-care therapies, could provide the opportunity of introducing highly active TRA-based combination therapies that might be broadly applicable in the cancer clinic in the future.
